# The Importance of Using Realistic 3D Canopy Models to Calculate Light Interception in the Field

**DOI:** 10.34133/plantphenomics.0082

**Published:** 2023-08-18

**Authors:** Shunfu Xiao, Shuaipeng Fei, Qing Li, Bingyu Zhang, Haochong Chen, Demin Xu, Zhibo Cai, Kaiyi Bi, Yan Guo, Baoguo Li, Zhen Chen, Yuntao Ma

**Affiliations:** ^1^ College of Land Science and Technology, China Agricultural University, Beijing, China.; ^2^ The State Key Laboratory of Remote Sensing Science, Aerospace Information Research Institute, Chinese Academy of Sciences, Beijing, China.; ^3^ Farmland Irrigation Research Institute of Chinese Academy of Agricultural Sciences/Key Laboratory of Water-Saving Agriculture of Henan Province, Xinxiang, China.

## Abstract

Quantifying canopy light interception provides insight into the effects of plant spacing, canopy structure, and leaf orientation on radiation distribution. This is essential for increasing crop yield and improving product quality. Canopy light interception can be quantified using 3-dimensional (3D) plant models and optical simulations. However, virtual 3D canopy models (VCMs) have often been used to quantify canopy light interception because realistic 3D canopy models (RCMs) are difficult to obtain in the field. This study aims to compare the differences in light interception between VCMs and RCM. A realistic 3D maize canopy model (RCM) was reconstructed over a large area of the field using an advanced unmanned aerial vehicle cross-circling oblique (CCO) route and the structure from motion-multi-view stereo method. Three types of VCMs (VCM-1, VCM-4, and VCM-8) were then created by replicating 1, 4, and 8 individual realistic plants constructed by CCO in the center of the corresponding RCM. The daily light interception per unit area (DLI), as computed for the 3 VCMs, exhibited marked deviation from the RCM, as evinced by the relative root mean square error (rRMSE) values of 20.22%, 17.38%, and 15.48%, respectively. Although this difference decreased as the number of plants used to replicate the virtual canopy increased, rRMSE of DLI for VCM-8 and RCM still reached 15.48%. It was also found that the difference in light interception between RCMs and VCMs was substantially smaller in the early stage (48 days after sowing [DAS]) than in the late stage (70 DAS). This study highlights the importance of using RCM when calculating light interception in the field, especially in the later growth stages of plants.

## Introduction

As the global population expands and climate extremes become more frequent and intense, feeding the world is an increasingly pressing challenge [1,2]. To address this challenge, plant scientists and breeders are striving to develop highly productive and resilient crops that can thrive under challenging environmental conditions [3,4]. Quantifying the impact of canopy light interception is critical to breeding high-yielding and resilient crops, with notable implications for global food security [5]. By understanding the effects of light interception on crop growth and yield, breeders can develop more efficient breeding strategies that prioritize traits that enhance yield potential [6]. This will lead to the development of more sustainable and resilient crops that can better withstand the effects of climate change [7].

Calculating the extent to which the canopy intercepts light is a crucial task in understanding plant growth and productivity. Over the years, researchers have devised various methods to tackle this complex problem. One of the most traditional ways of measuring light interception is through the use of photosynthetically active radiation (PAR) sensors. This approach, although relatively primitive, is still a go-to option for model validation. On the other hand, experts prefer to estimate light interception by calculating the leaf area index (LAI) of the canopy and using extinction coefficients [8,9]. However, in recent years, scientists have been exploring more innovative approaches such as utilizing 2-dimensional (2D) images for the inversion of light interception [10,11], while the intricate spatial arrangement of canopy components like leaf clumping, gaps, and variations in leaf angles still pose a steep challenge for researchers [12]. Despite these difficulties, the development of new and improved methods for analyzing light distribution within a canopy remains critical for advancing our understanding of plant physiology and ecosystem processes.

The pursuit of detailed light analysis in 3-dimensional (3D) spaces through the use of optical simulation methods has emerged as a crucial avenue of research, with radiation transfer model being a prominent technique employed in virtual plant models [13,14]. Radiation transfer model has proven to be an extraordinarily effective method for computing the interaction of light with complex plant canopies [15]. By tracing the paths of individual light rays, the technique facilitates the meticulous analysis of their multifaceted interactions with canopy leaves, encompassing absorption, reflection, and refraction phenomena [16,17]. This intricate interplay enables the accurate quantification of the amount of light intercepted by individual leaves, as well as the canopy as a cohesive unit. Therefore, the use of radiation transfer techniques has contributed greatly to the better understanding of the interplay between light and plant structures in 3D spaces.

Functional–structural plant models (FSPMs), one of the virtual plant models, simulate plant growth and development by combining 3D architectural information with physiological processes (e.g., respiration, photosynthesis, transpiration, and nutrient uptake) [18,19]. FSPMs can be classified as rule-based (dynamic) or static, with the former focusing on the dynamic growth processes of plants while the latter can describe the structural details of plants at specific growth stages [20]. The integration of radiation transfer model and FSPMs has been utilized to study changes in light interception caused by plant structure variations [7,21]. However, the lack of detailed descriptions of the organ level (leaf veins and leaf folds) in the available FSPMs can limit the accuracy of assessing the actual light cutoff of plants [13]. To achieve a more realistic representation of plant structure, several sensor-based 3D technologies have been developed. These technologies, such as handheld laser scanning [22], structured light [23], Light Detection and Ranging (LiDAR) [24], and time of flight [25], enable the acquisition of accurate 3D canopy models. These realistic 3D plant canopy models (RCMs) combined with the radiative transfer model allow for accurate calculations of canopy light interception and hence photosynthetic efficiency [13].

Efficient and accurate acquisition of the 3D structure of plant canopies in the field is a prerequisite for calculation of light interceptions. However, existing 3D techniques have limitations in capturing 3D canopy structure in the field [26]. For example, structured light and time-of-flight cameras are much less accurate in direct sunlight [27] and laser scanning is inadequate to measure the complete 3D structure of the canopy [28]. In order to calculate the light distribution of a field canopy, researchers typically generate an RCM with a single plant or multiple 3D plants (e.g., 4 plants) and then replicate it as a complete virtual canopy [29,30]. However, it has not been reported whether these virtual canopies can replace real plants grown in the field for light interception calculation.

Structure from motion (SfM) is a cost-effective 3D reconstruction method that utilizes a set of overlapping images to generate a 3D model of an object [26,31]. This technique only requires an RGB camera, making it an accessible alternative to 3D sensor-based methods. The combination of SfM methods with drones has provided an economical and convenient system for estimating plant traits, such as plant height [32] and LAI [33]. Recently, a new approach, the “cross-circling oblique (CCO) route”, has been introduced that utilizes drones and SfM to reconstruct accurate 3D canopies in the field and to estimate organ-level characteristics, such as leaf length and width, with high precision [34,35]. This method sheds new light on the study of light interception in the field canopy.

In this study, an RCM of maize in the field was reconstructed using an unmanned aerial vehicle (UAV) and SfM based on a UAV CCO route. A corresponding virtual 3D canopy (RCM) (64 plants) was constructed based on the replications of single, 4, and 8 realistic plants, respectively. The objectives of our study were as follows: (a) to reconstruct an RCM of field maize based on the UAV CCO route and verify its accuracy, and (b) to verify the differences in the distribution of light interception between the RCM and the different virtual canopy models (VCMs) in the field based on a radiation transfer model.

## Materials and Methods

### Experimental design

#### Workflow

The RCM was reconstructed using the SfM algorithm based on the UAV CCO route. In addition, VCMs were generated from 1, 4, and 8 precise structures of individual plants, designated VCM-1, VCM-4, and VCM-8, respectively. Light interception was then used to compare the RCM and VCMs under identical simulation conditions. Finally, the multidimensional phenotypic traits of the RCM and VCMs were extracted to analyze the variations between the 2 canopies.

#### Experimental setup and study area

**Fig. 1.**
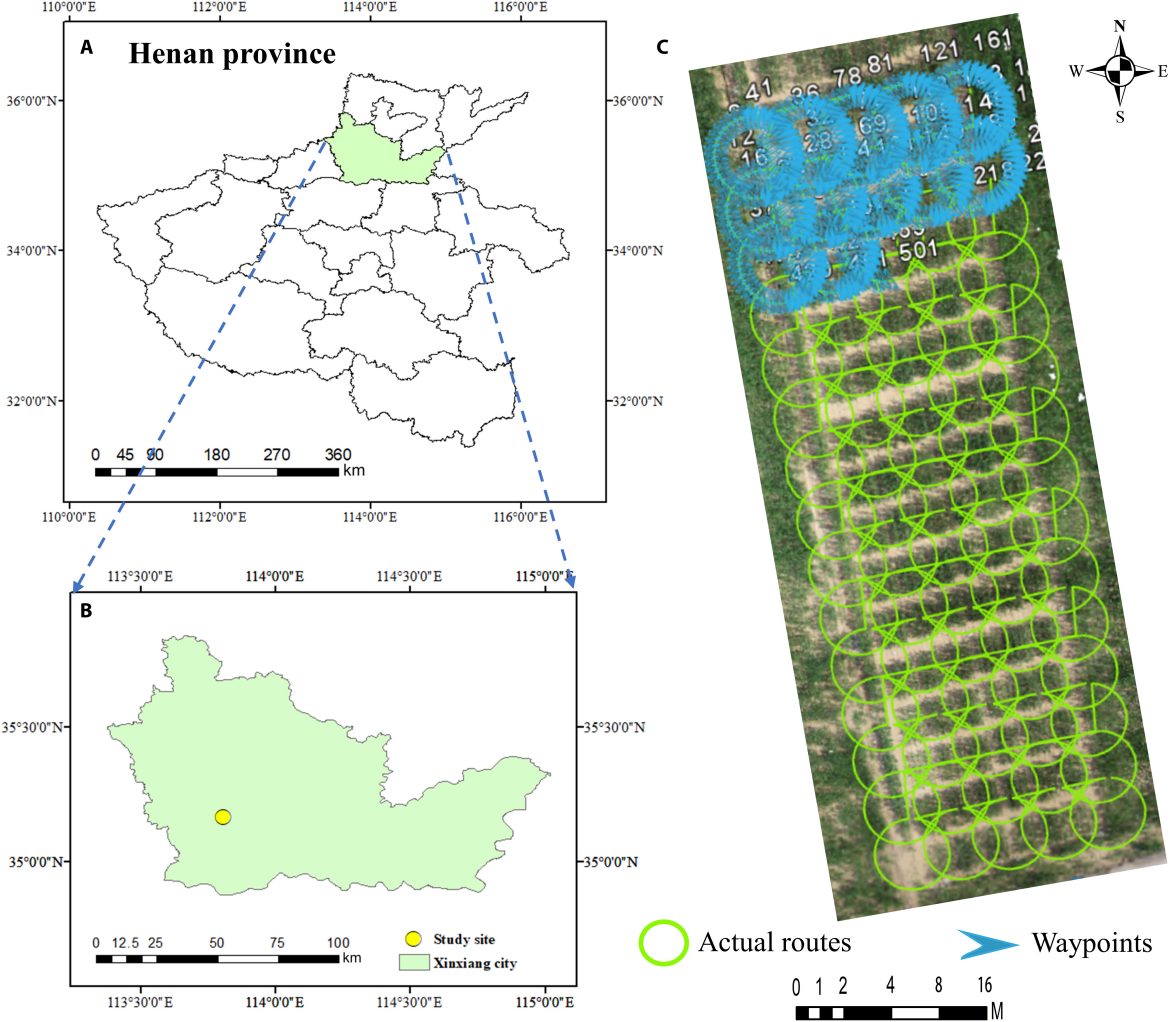
(A) Location of the city where the study area is located within the province; (B) location of the study site in the city; (C) the orthomosaic image acquired on 2022 August 12 and actual CCO flight paths for 90 plots. The green circle represents the drone's flight path, the blue arrow signifies the drone's waypoints, and the white digits show the waypoint number. To provide a clear view of the full path, only a portion of the drone's waypoints and their corresponding numbers are illustrated.

The study site is situated at the Institute of Agricultural Land Irrigation, Chinese Academy of Agricultural Sciences, Xinxiang, Henan, China (11°51′ E, 35°18′ N). This experimental site is located in the northern plain of Henan Province, south of the Yellow River, as shown in Fig. 1. Henan has a warm temperate and subtropical monsoon climate with a variety of distinct seasons. The winters are cold and generally dry, with little rain and snow. The springs are short and dry, with a lot of sand. The summers are hot and rainy, while the autumns are long and sunny.

From June to September 2022, a trial was conducted using 90 plots of 4 × 2 m^2^ each. The spacing between the plants was 25 cm, and the spacing between the rows was 66 cm. Ten different varieties of maize were planted. The study applied 3 levels of N fertilizer (0, 80, and 120 kg/ha) and replicated each plot 3 times. Buffer strips were set up between treatments to avoid mutual effects. On 2022 June 15, 192 seeds were sown in each plot, and 64 seedlings with good growth were retained after emergence.

### Realistic 3D canopy model reconstruction

#### UAV data collection

**Fig. 2.**
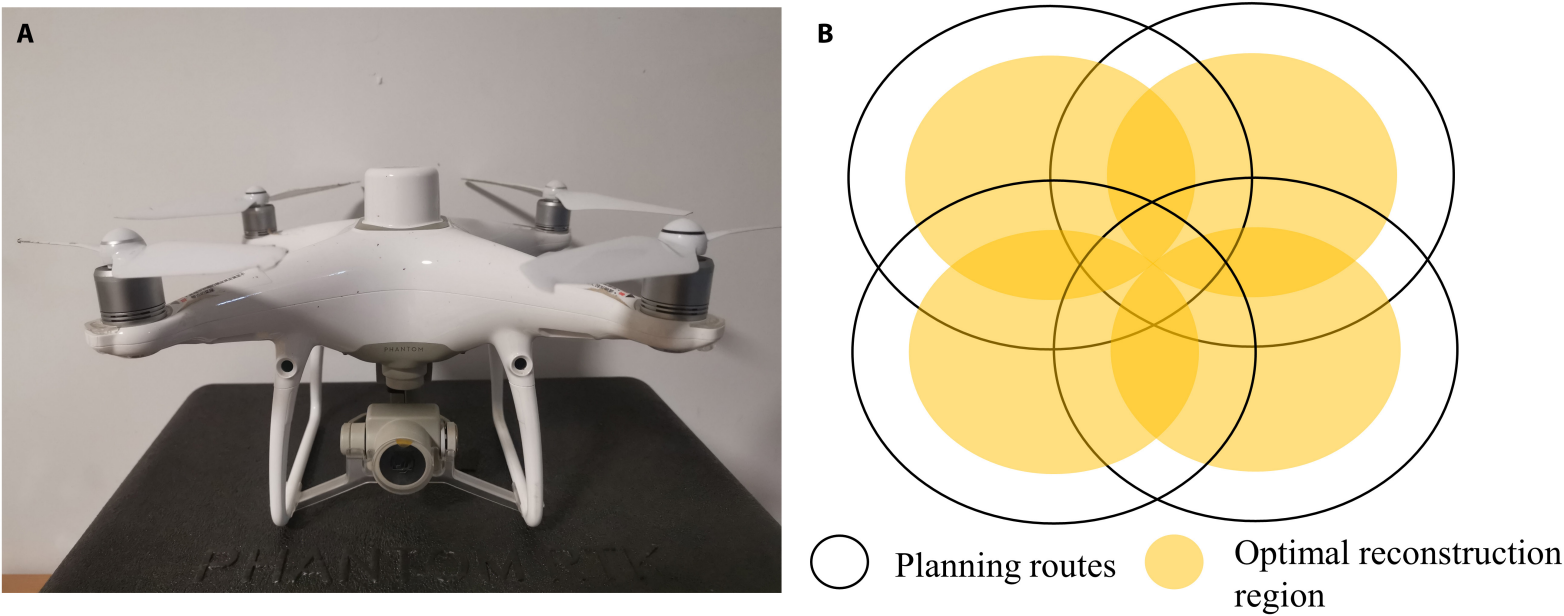
Diagram of the cross-circling oblique (CCO) route. (A) DJI Phantom 4 RTK drone. (B) CCO route consisting of 4 single circles with a 50% inter-circle overlap.

The technique of the CCO route involves taking multiple single-circle routes, as shown in Fig. 2B. The overlap in the CCO route is classified as intra-circle overlap and inter-circle overlap. Intra-circle overlap pertains to the overlap between images within a single circle and is determined by the camera's field of view, the radius of the circle, and the altitude of the flight. On the other hand, inter-circle overlap refers to the overlap between adjacent single-circle routes and affects the overlap between images of adjacent intra-circle routes. Figure 2B illustrates a CCO route comprising 4 single circles with a 50% inter-circle overlap, where the yellow areas represent the optimal reconstruction region.

The UAV image sequences were acquired on a windless day. Two data collection sessions for maize were carried out via the CCO route, specifically on 2023 August 3 and 25. The flight altitude for the CCO route was adjusted to be 4 to 5 m above the canopy, with actual adjustments made depending on the height of the canopy. The circle radius was set to 4 m, and the camera angle was set to 45°. The route overlap was set to approximately 90% intra-circle overlap and 60% inter-circle overlap. The flight speed was 5 m/s. The interval of captured images was 0.2 s.

The DJI Phantom 4 RTK drone (Fig. 2A) was used to collect data for the CCO route (Fig. 2C) over the target area. The drone is a small and lightweight commercial platform (1,391 g) equipped with a high-resolution RGB camera (FC6310R, DJI, China; 5,472 × 3,648 pixels; Table 2). The CCO route was generated automatically using Waypoint Master software (Weber Intelligent Control Technology Ltd, Beijing, China) by entering the geographic extent of the target area as Keyhole Markup Language (KML) and a digital surface model (DSM). After setting the flight parameters, the software generated a mission file that was then imported into the remote control. The CCO route could then be invoked for automatic flight. The 2 DJI Phantom 4 RTK drones took a total of half an hour to complete the data collection. After UAV data collection, the length and width of labeled maize leaves at different layers were manually measured to verify the accuracy of reconstructed canopy models.

#### Reconstruction of 3D canopies and pre-processing

To reconstruct 3D models of maize canopies, UAV image sequences were processed using Agisoft Metashape Professional (Agisoft LLC, St. Petersburg, Russia; version 1.7.3). The standard SfM-multi-view stereo (MVS) workflow was used to identify image keypoints and to assign keypoint descriptors, which were then used to estimate camera parameters and positions. Agisoft Metashape software also used accurate latitude, longitude, and elevation information from the images to scale the point cloud correctly. The software then used the internal and external camera orientation parameters to obtain a sparse point cloud. In order to acquire high-density and precise point clouds for vegetation, we utilized the “high setting” model that incorporated image alignment options and dense cloud construction. This approach has the capability to augment the point density by over 10 times. Finally, the MVS method was used to achieve dense point cloud reconstruction.

The point cloud data were pre-processed using slope-based [36] and statistical filtering [37] techniques implemented in Python (version 3.5, http://www.python.org) with the Open3D library [38]. To segment the data into individual plots, a 2-step process was employed. Firstly, the 4 corner coordinates of the entire study region (30 plots) were manually identified. Using the simple features [39] and raster [40] packages in R, version 4.0 [41], 30 spatial polygons were established, corresponding to each plot evenly. The dense point cloud was then segmented into 30 plots using these polygons and the Whitebox Tools library [42].

Point clouds of all plots were further generated canopy surface to compute light interception. The voxel-based method [43] with a ratio of 0.02 was used to downsample the 3D canopy point clouds to improve the efficiency and accuracy of the facets. The downsampled canopy point clouds were triangulated using the improved Crust algorithm [34]. The original Crust algorithm [44] computes the Delaunay triangulation of the point set, constructs the “crust” of the triangulation, and prunes the crust to obtain a set of edges. The resulting edges are triangulated to create the surface mesh. The algorithm can handle noisy and incomplete data, but the canopy triangulation model built with the Crust algorithm contained numerous abnormal facets. To address this issue, the improved Crust algorithm used the interquartile range method to remove abnormal facets.

#### Validation of RCM

The accuracy of the reconstructed canopy models was confirmed through the estimation of the length and width of the leaves, followed by comparison with manual measurements. The first step involved the segmentation of the point cloud of labeled leaves from the canopy point cloud. Then, the point clouds were meshed. After that, the length and width of the leaves were manually estimated using the Geomagic Studio 2013 software (3D Systems, Inc., South Carolina, USA). The leaf length was determined as the surface distance from the base to the tip of the leaf, while the leaf width was estimated as the maximum surface distance perpendicular to the leaf length. Finally, the estimated traits and manual measurements were compared using coefficients of determination (*R*^2^), root mean square error (RMSE), and relative root mean square error (rRMSE) (Eqs. 1 to 3).

$$R^{2}=1-\frac{\sum_{i=1}^{n}{\left(y_{i}-\hat{y_{i}}\right)}^{2}}{\sum_{i=1}^{n}{\left(y_{i}-\bar{y_{i}}\right)}^{2}}$$ (1)

$$\text{RMSE}=\sqrt{\frac{1}{n}\sum_{i=1}^{n}{\left(y_{i}-\hat{y_{i}}\right)}^{2}}$$ (2)

$$\text{rRMSE}=\frac{\text{RMSE}}{\bar{y_{i}}}\times 100\%$$ (3)

where *n* is the number of validation samples, *y_i_* is the manually measured value, $\hat{y_{i}}$ is the estimated value, and $\bar{y_{i}}$ is the mean value of the manually measured value.

### Virtual 3D canopy model generation

**Fig. 3.**
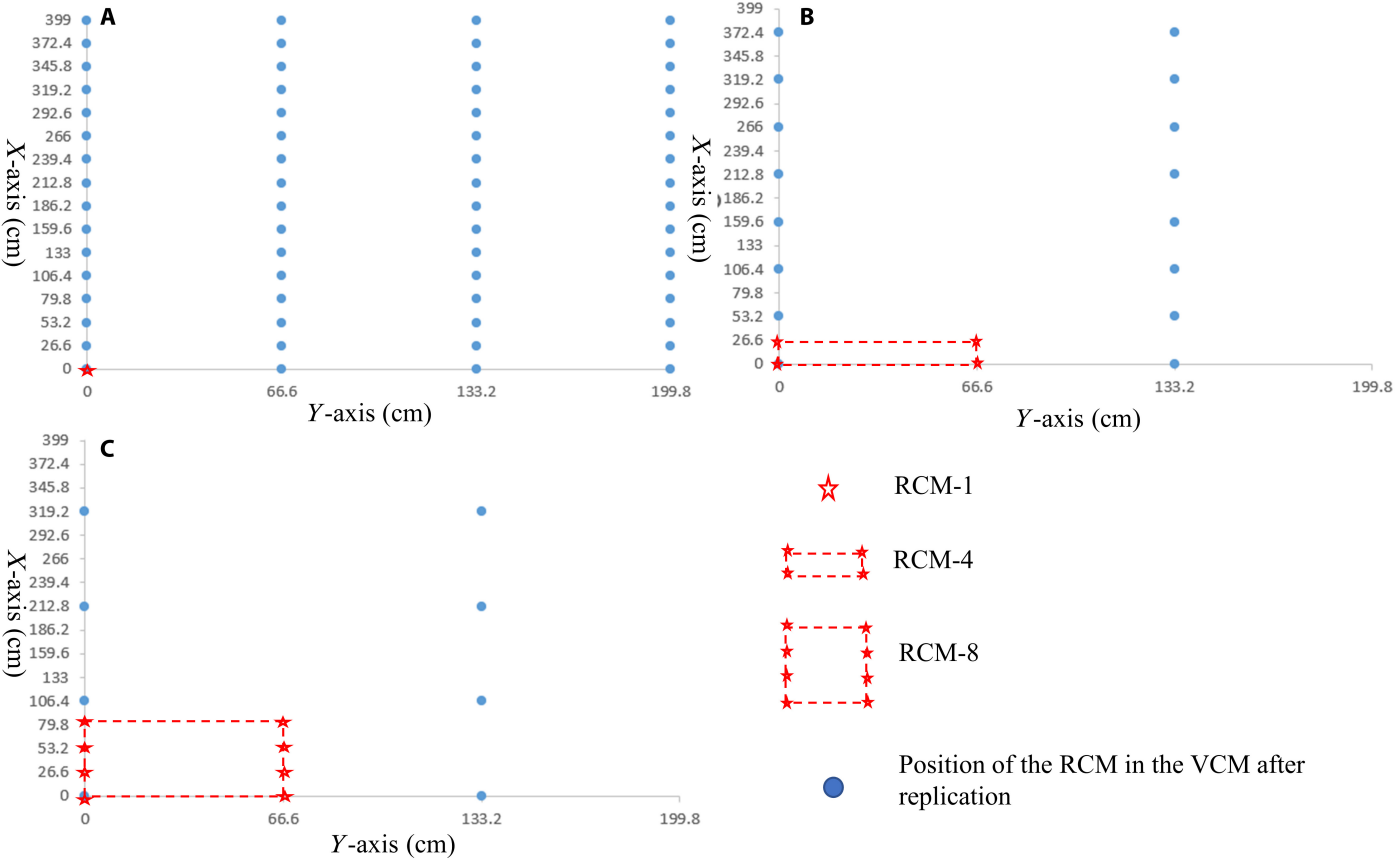
Schematic diagram of virtual canopy model generation with 64 maize plants. (A) Virtual canopy model (VCM-1) generation using a single maize plant of a realistic 3D model (RCM-1); (B) virtual canopy model (VCM-4) generation using 4 maize plants of a realistic 3D model (RCM-4); (C) virtual canopy model (VCM-8) generation using 8 maize plants of a realistic 3D model (RCM-8).

To compare the difference in light interception between the RCM and VCMs, artificial plant segmentation of 1 plant (RCM-1), 4 plants (RCM-4), and 8 plants (RCM-8) was carried out in the middle of the RCM. The plants in the middle of the RCM were selected for the construction of the VCMs because the CCO-derived 3D maize canopy was also better reconstructed in the middle region (Fig. S1). In addition, the plants in the side rows were sometimes unusually tall, which could cause errors in the VCMs. These were then replicated into VCMs with 64 plants to obtain 3 types of VCMs, namely, VCM-1, VCM-4, and VCM-8. Using CloudCompare software, 1, 4, and 8 plants were manually divided from the RCM in the middle. The RCM has 64 plants with a row spacing of 66.6 cm and a plant spacing of 26.6 cm. For a more accurate comparison, the same plants and row spacing were used for the VCMs. Prior to replication, the coordinates of the positions of the single, 4, and 8 realistic 3D canopies replicated into the VCMs were calculated (blue dots in Fig. 3). In the construction of VCM-1, RCM-1 was replicated 64 times and moved to the corresponding positions. In the construction of VCM-4, RCM-4 was copied 16 times and moved to the corresponding positions. Finally, RCM-8 was copied 8 times and moved to the appropriate position to build VCM-8.

### Light interception calculation and comparison between VCMs and RCM

The solar radiation distribution was calculated in the Helios 3D modeling framework [45], which uses Bailey's radiation model. 3D canopy triangle models were uploaded into the radiation model in ply format. The reverse ray tracing method was employed in this radiative transfer model [46]. PAR (400 to 700 nm) was calculated based on this model. The properties of each band were simplified to be consistent; i.e., the leaf reflectance and transmittance for each band were set to 0.1. The canopy light distribution was simulated from 0600 h to 1800 h for each measurement date at 1-h intervals. Scattering iterations was set as 8. Periodic boundary condition was set to eliminate domain edge effects. Direct and diffuse solar fluxes were calculated using the REST-2 model [47]. REST-2 is a state-of-the-art atmospheric transmission model that calculates the solar flux at Earth's surface after attenuation by water vapor, CO_2_, ozone, NO_2_, and aerosols. The model partitions the total radiative flux into direct and diffuse components. The position of the sun at different moments was calculated from the description in Ref. [48]. The REST-2 model and the sun position model were already implemented in the 3D model Helios. All input parameters are summarized in Table S1.

First, the light interception per square meter per face (W/m^2^) was simulated. After simulations, the daily light interception per unit area (DLI; Eq. 4) was calculated.

$$\mathrm{DLI}=\frac{\int_{t=6}^{18}\left(\sum_{i=1}^{m}s_{i}*{\mathrm{LF}}_{i}\right)\mathrm{dt}}{S}$$ (4)

where *LF_i_* is the captured radiation of facet L, *s_i_* is the area of facet L, *m* is the number of facets, and *S* is ground area.

To assess the light distribution on various dates of measurement, we employed a method that involves calculating the ratio of daily light interception at each horizontal layer, termed LR. This is illustrated in Eq. 5. In this context, DLI_h_ denotes the daily light interception at each individual horizontal layer, which, in total, was divided into 10 layers.

$$\mathrm{LR}={\mathrm{DLI}}_{h}/\mathrm{DLI}$$ (5)

### Multidimensional phenotypic trait calculation and comparison between VCMs and RCM

To further analyze the reasons for the differences in light interception between VCMs and RCM, phenotypic parameters were extracted from 3 dimensions for comparative analysis. Canopy cover and plant height were chosen as one-dimensional phenotypic parameters to represent the horizontal and vertical characteristics of the canopy, respectively. LAI, which is often used in studies of canopy leaf area distribution, was chosen as the 2D phenotype. Canopy occupation volume (COV), a parameter that effectively explains the joint effect of leaf area and leaf inclination on photosynthesis [49], was selected as the 3D phenotype.

The distance between the lowest point of the plant and the 99th percentile height was used to define plant height. To calculate canopy coverage, the canopy occupation area was divided by the projected canopy area. Projected canopy area was calculated using alphashape [50]. To calculate LAI, the total area of leaves was divided by the ground area. Total leaf area of the canopy was calculated from meshed canopy using Heron's formula [51]. The canopy model was decomposed into several voxels (1 cm × 1 cm × 1 cm) to calculate COV. Only the voxels that contained the triangles of the canopy model were considered as occupied voxels. The COV was defined as the total volume of these occupied voxels. The above steps were all implemented using Python, version 3.5 (http://www.python.org).

## Results

### Accuracy verification of 3D reconstruction models

**Fig. 4.**
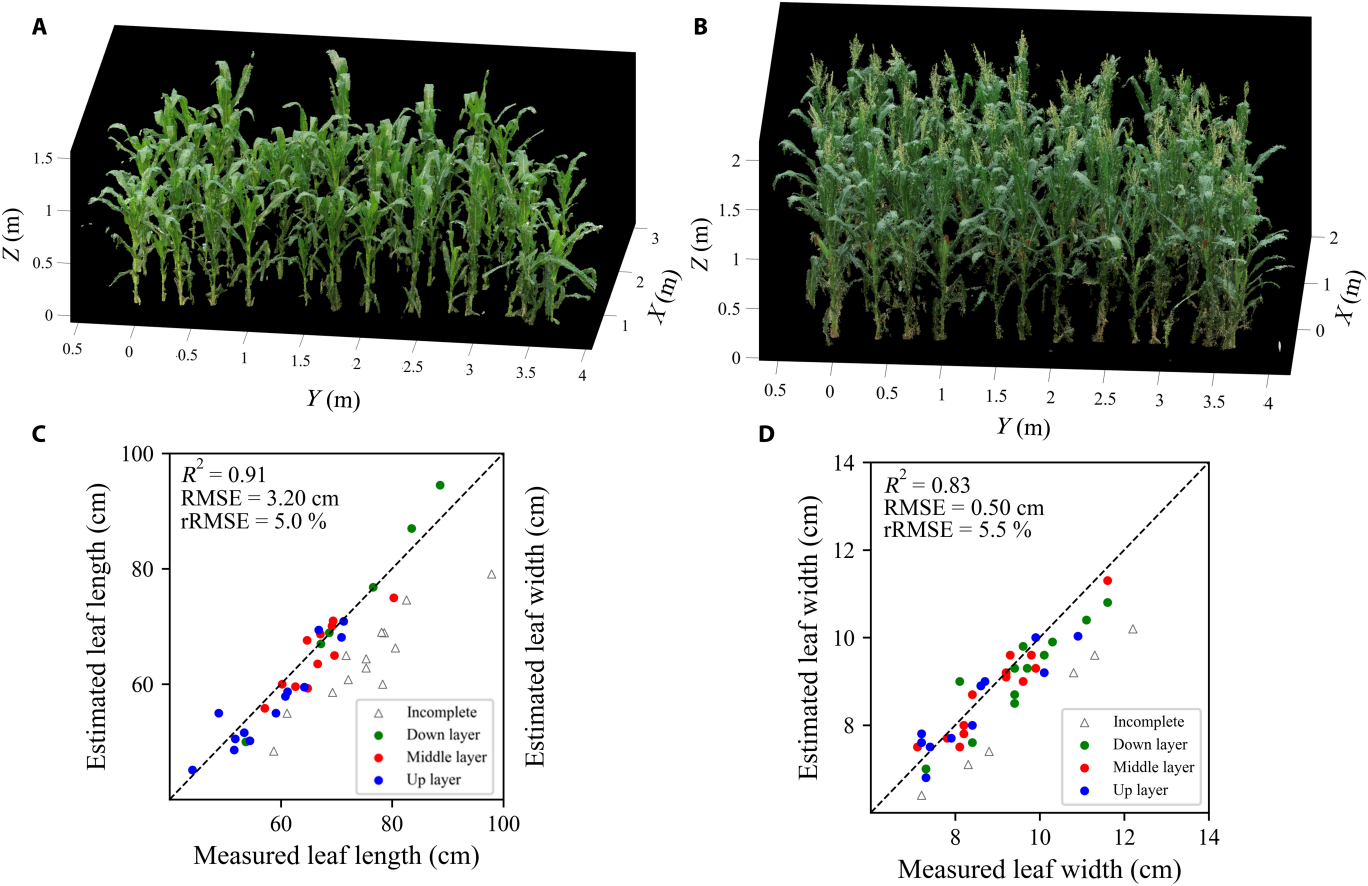
3D reconstruction model accuracy verification. Incomplete in the legend indicates incomplete leaves after 3D reconstruction. (A) 3D reconstruction maize canopy at 48 DAS; (B) 3D reconstruction maize canopy at 70 DAS; (C) scatter plot of estimated marked leaf length against measured values; (D) scatter plot of estimated marked leaf width against measured values.

The reconstructed 3D point clouds of maize provide highly detailed and realistic visualizations of the plant structure, enabling a comprehensive assessment of the plant's growth and development. The reconstruction of the maize canopy with well-defined leaf veins and the clear visibility of the maize tassel in the side view, as demonstrated in Fig. 4A and B, provide insights into the plant's anatomy and physiology. Quantitative analysis was performed to validate the accuracy of the obtained data. During the analysis, estimated leaf length and leaf width were compared with the measured values. Results showed high accuracy of the estimated values for both periods with *R*^2^ values of 0.91 and 0.83 for leaf length and leaf width. Moreover, the low RMSE values of 3.20 cm and 0.83 cm for leaf length and leaf width and rRMSE values of 5.0% and 5.5% for leaf length and leaf width, respectively, demonstrated the precision of the CCO approach (Fig. 4C and D).

### Comparison of light interception between RCM and VCMs

**Fig. 5.**
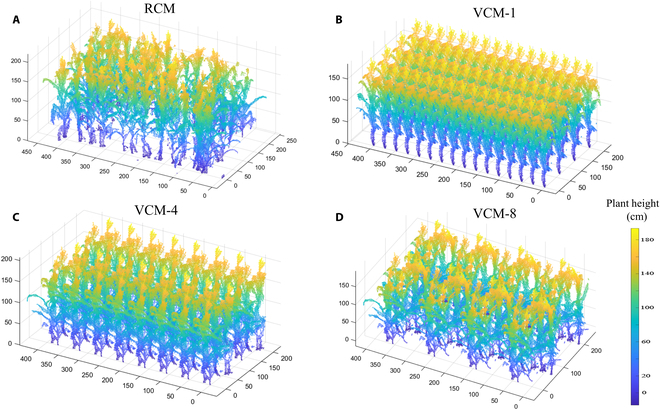
Schematic representation of the realistic 3D canopy model (RCM) and the virtual canopy model (VCM). (A) 3D reconstructed maize canopy by cross-circling oblique (CCO) route. Virtual 3D canopy replicated with a single maize plant (B), with 4 maize plants (C), and with 8 maize plants (D).

After downsampling, the RCM and VCMs were generated for all plots with 2 periods (48 and 70 days after sowing [DAS]). A schematic diagram of the RCM and VCM of a plot from 70 DAS is shown in Fig. 5. VCM-1, VCM-4, and VCM-8 were replicated from the corresponding RCM middles 1, 4, and 8 plants, respectively. Intuitively, it appears that as the number of replicated plants increases, the corresponding VCMs become closer and closer to the RCM.

**Fig. 6.**
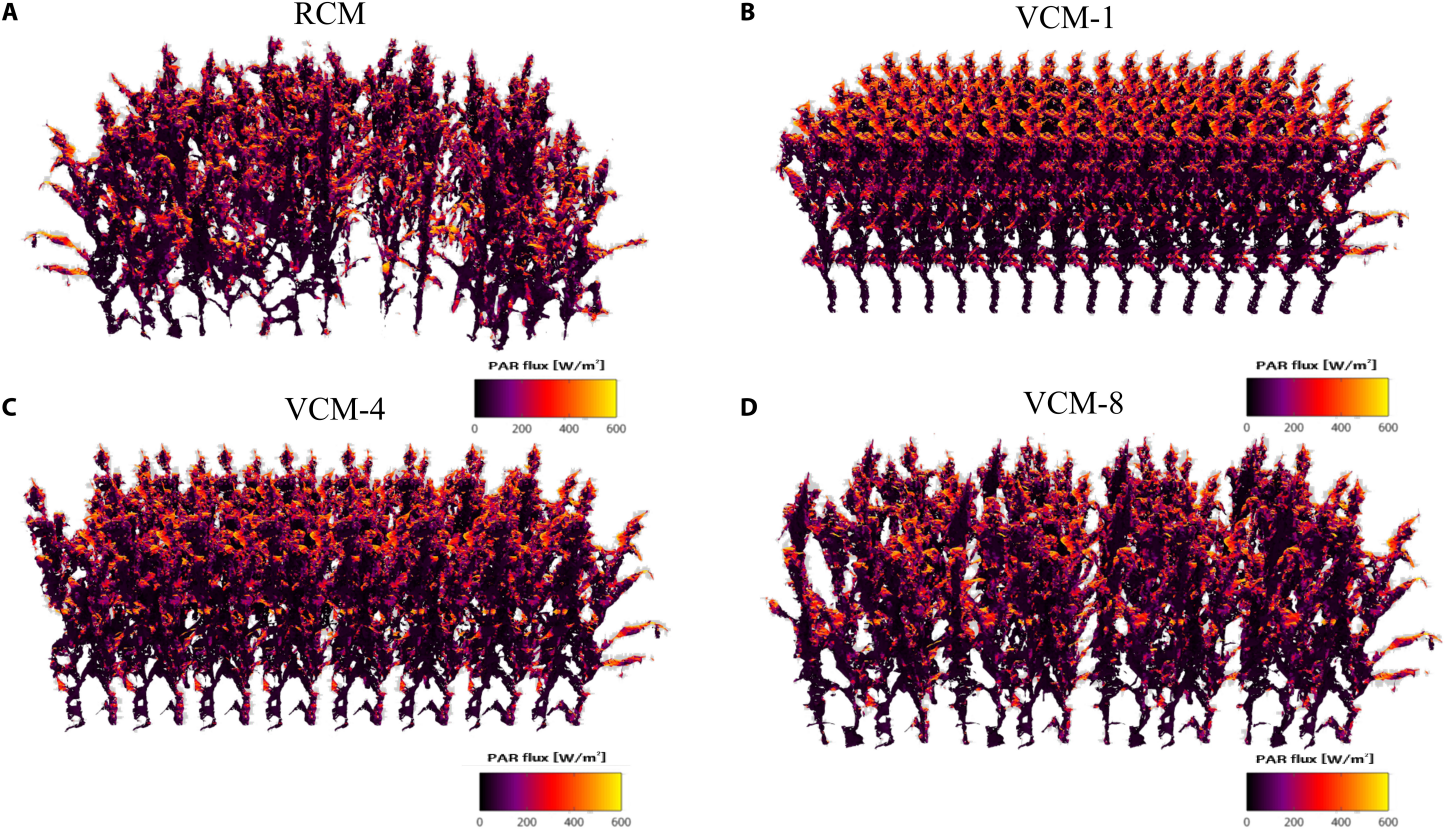
The visualization of light capture of (A) realistic 3D canopy model (RCM), (B)virtual 3D canopy replicated with a single maize plant VCM-1, (C) virtual 3D canopy replicated with 4 maize plants VCM-4, and (D) virtual 3D canopy replicated with 8 maize plants VCM-8, from side views at 70 days after emergence at 1200 h.

**Fig. 7.**
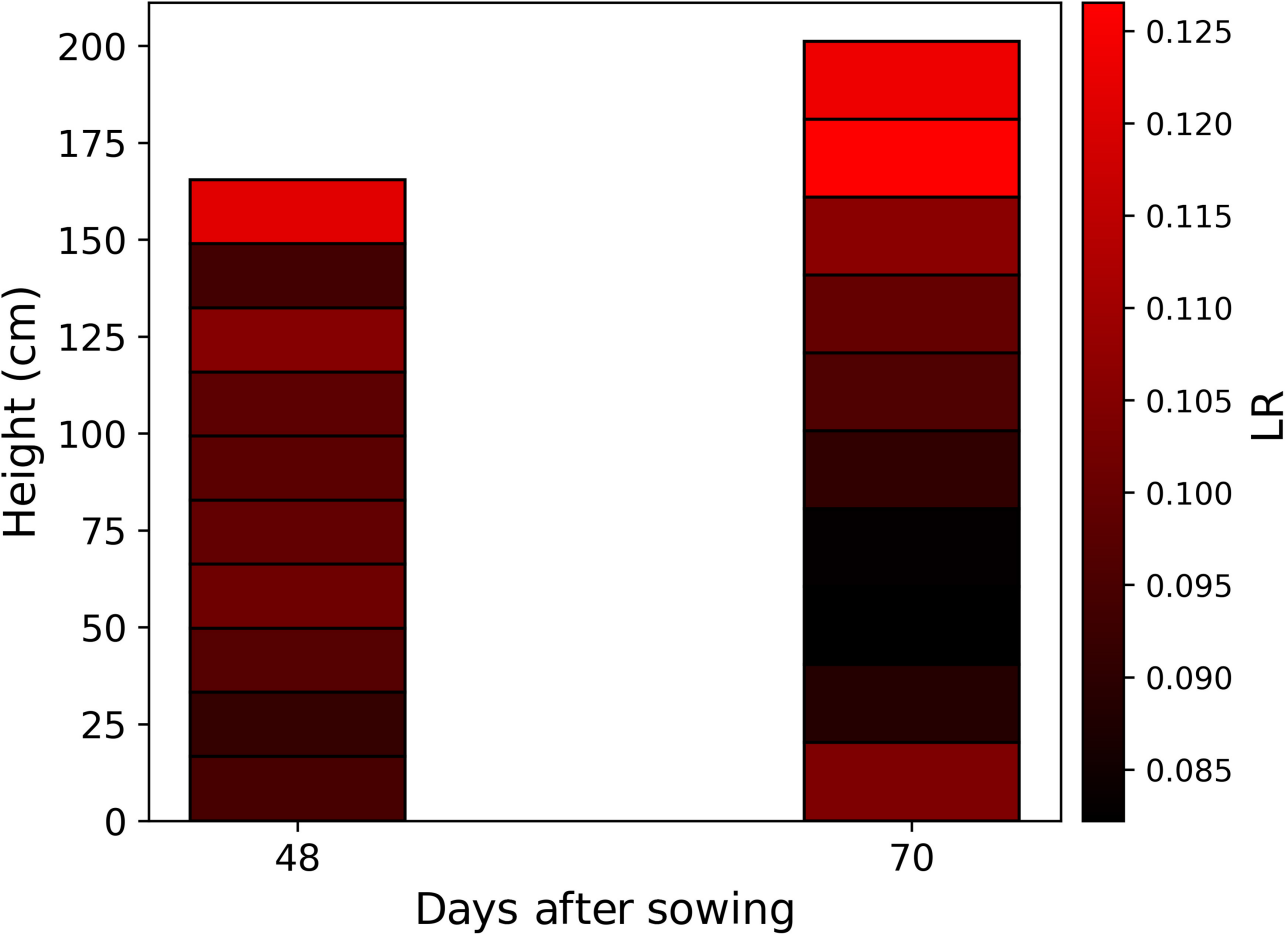
The light distribution of RCM at 48 and 70 days after sowing. LR represents the ratio of daily light interception at each horizontal stratum.

Figure 6 visualized the light interception within the different canopies (RCM and VCM) by coloring the value of the captured light radiation. Higher light interception is indicated by more yellow colors, while lower light interception is indicated by darker black colors. In RCM (Fig. 6A), radiation is concentrated in the middle and upper part of the canopy on the left, where plants are more densely distributed. The lower layers receive less radiation. On the right side, where the plants are more scattered, the lower leaves also intercept some radiation (Fig. 6A). For VCM-1 (Fig. 6B), the plants are closely spaced and the radiation is mainly concentrated at the top of the canopy, with very little light penetrating the lower part of the canopy. For VCM-4 (Fig. 6C) and VCM-8 (Fig. 6D), the plants are more sparsely distributed, with most radiation in the upper part of the canopy and some in the middle and lower parts. Vertical stratification of canopy light capture for 2 periods is also mapped in Fig. 7. At 48 DAS, the vertical layer exhibited a more uniform light distribution due to the smaller shading of the canopy. However, by 70 DAS, the upper layer had intercepted most of the light, while the middle and lower layers received little light interception due to substantial canopy shading.

DLI was calculated for both RCM and VCMs at 2 growing stages (48 and 70 DAS) and compared in Table 1, and the scatter plot is shown in Fig. S3. The correlation between the DLI estimated by the RCM and the VCMs increased with the number of replicates. VCM-1 had the lowest correlation with the DLI estimated by RCM, with an *R*^2^ of 0.45, an RMSE of 3.92 (MJ m^−2^ s^−1^) and an rRMSE of 20.22%. VCM-4 had a weak correlation with the DLI estimated by RCM, with an *R*^2^ of 0.60, an RMSE of 3.36 (MJ m^−2^ s^−1^), and an rRMSE of 17.38%. VCM-8 had the relatively high correlation with the reconstructed canopy, with an *R*^2^ of 0.68. However, the rRMSE was still greater than 10% (15.48%). This demonstrates the importance of analyzing light interception by constructing a fully realistic 3D canopy in the field. It was also found that the correlation between the DLI of VCM and RCM was higher at 48 DAS than at 70 DAS (nRMSE of 18.30% vs. 22.59% for VCM-1, nRMSE of 15.79% vs. 18.19% for VCM-4, and nRMSE of 13.83% vs. 16.34% for VCM-8).

**Table 1.** Comparison of daily light interception per unit area (DLI) between VCM and RCM in different growing stages.

	VCM-1 vs. RCM		
	All-stage	48 DAS	70 DAS
*R* ^2^	0.45	0.61	−0.76
RMSE (MJ m^−2^ s^−1^)	3.92	3.91	3.92
rRMSE	20.22%	22.59%	18.30%
	VCM-4 vs. RCM		
	All-stage	48 DAS	70 DAS
*R* ^2^	0.60	0.81	−0.74
RMSE (MJ m^−2^ s^−1^)	3.36	2.73	3.89
rRMSE	17.38%	15.79%	18.19%
	VCM-8 vs. RCM		
	All-stage	48 DAS	70 DAS
*R* ^2^	0.68	0.85	−0.4
RMSE (MJ m^−2^ s^−1^)	3.0	2.4	3.5
rRMSE	15.48%	13.83%	16.34%

**Fig. 8.**
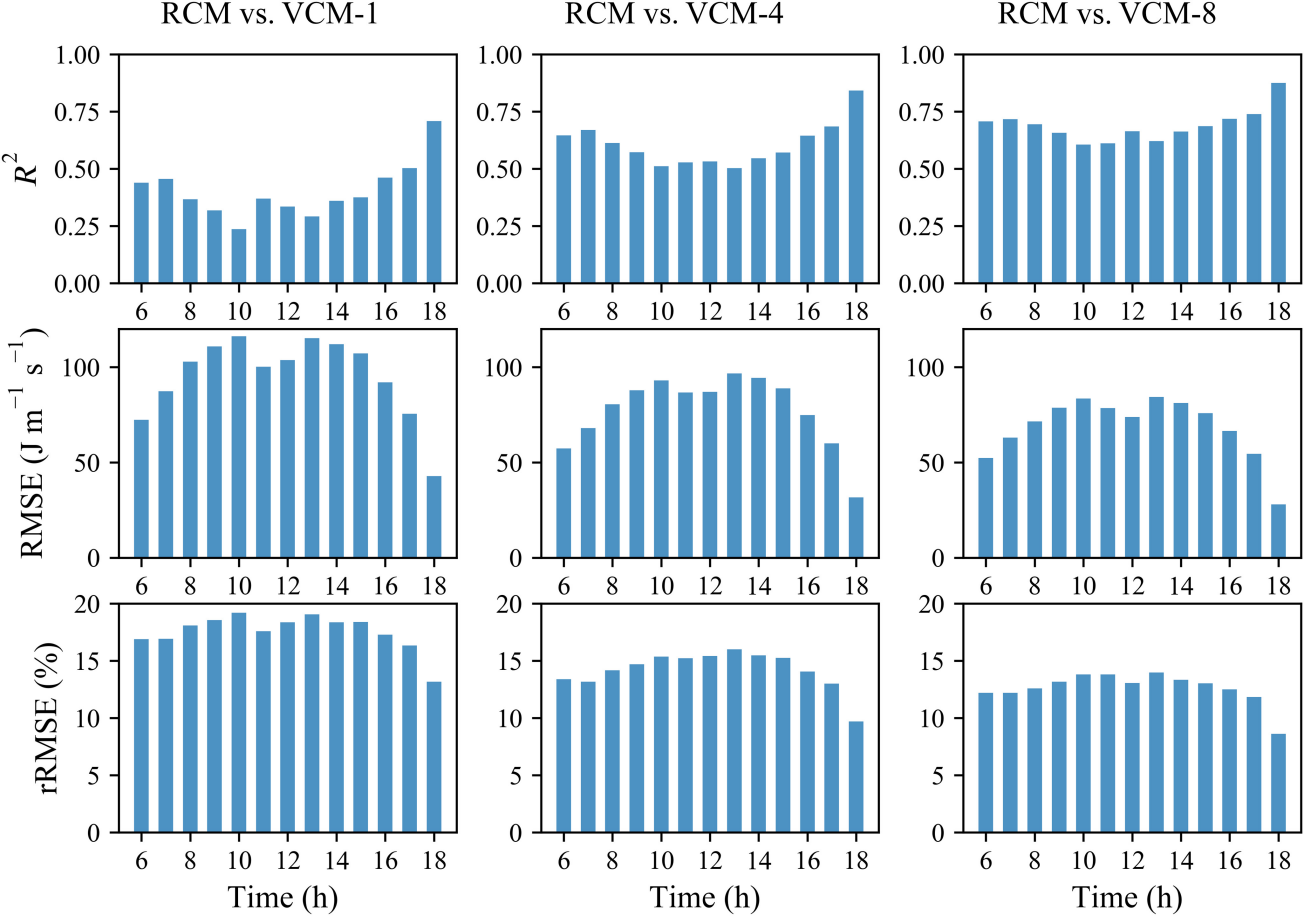
Comparison of hourly histogram of light capture error between the realistic 3D canopy model (RCM) and the virtual canopy model (VCM) at 2 stages. VCM-1, VCM-4, and VCM-8 represent the VCM replicated with a single maize plant, 4 maize plants, and 8 maize plants from RCM, respectively.

Hourly light capture error comparison between the RCM and VCMs at 2 stages is shown in Fig. 8. At 6 to 17 hours, there was weak correlation of hourly light capture between RCM and VCM-1 (*R*^2^ = 0.38), with mean RMSE and rRMSE values as high as 99.60 (J m^−2^ s^−1^) and 17.92%, respectively. A relatively strong correlation was observed for light capture between RCM and VCM-1 only at 1800 h with an *R*^2^ of 0.70, an RMSE of 43.00 (J m^−2^ s^−1^), and rRMSE of 13.15%. There was a relatively strong correlation for light capture between RCM and VCM-4 throughout the day with an *R*^2^ of 0.60, an RMSE of 77.43 (J m^−2^ s^−1^), and an rRMSE of 14.22%. There was a strong correlation for light capture between RCM and VCM-8 throughout the day with an average *R*^2^ of 0.69 and an average RMSE of 68.60 (J m^−2^ s^−1^). However, the average rRMSE was still greater than 10% (12.62%), indicating that a more realistic canopy is still required, although VCM-8 can explain the hourly light capture of RCM to a certain extent. Hourly light capture within the RCM and VCMs at 2 stages is also plotted in Fig. S2. The hour-by-hour light intercept curve is M-shaped, which is due to the fact that the corn was planted in a north–south direction. At midday, the sunlight will be nearly parallel to the planting direction, and the front crop will shade the back crop more heavily, thus reducing light interception [45,52].

### Comparison of multidimensional phenotypic traits between RCM and VCMs

The canopy structure can directly influence the distribution of light interception; thus, the structural differences between the RCM and 3 VCMs were analyzed (Table 2). It can be observed that as the canopy density increases, the 1D phenotypic (plant height and canopy cover) differences between VCM and RCM diminish. However, the differences in the 2D and 3D phenotypes (plant area index and COV) between VCM and RCM increase. This result illustrated that the difference in canopy structure between VCM and RCM is increasing with increasing canopy density, as the 2D and 3D phenotypic parameters better portray the canopy structural changes compared to the 1D phenotypic parameters. As the number of replications increased, the structural differences between the RCM and the VCMs decreased (Table 2). However, for both 2D PAI and 3D COV traits, the differences between the VCMs and the RCM were marked, with low *R*^2^ values (<0.3) and high rRMSE values (>30%). In addition, the VCMs had higher estimates than the RCM. The one-dimensional horizontal traits CC showed large variations, with the smallest variation (VCM-8) having an *R*^2^ of only 0.43 and an rRMSE of 31.92%. In contrast, the one-dimensional vertical traits PH showed little difference between VCM-8 (*R*^2^ = 0.85, RMSE = 13.16, rRMSE = 7.4%), VCM-4 (*R*^2^ = 0.73, RMSE = 17.81, rRMSE = 10.02%), and RCM.

**Table 2.** Comparison of multidimensional phenotypic traits, including canopy cover (CC), plant height (PH), plant area index (PAI), and canopy occupation volume (COV), estimated from 3D reconstructed canopy (RCM) and virtual 3D canopy (VCM).

CC									
	VCM-1 vs. RCM			VCM-4 vs. RCM			VCM-8 vs. RCM		
	All-stage	48 DAS	70 DAS	All-stage	48 DAS	70 DAS	All-stage	48 DAS	70 DAS
*R* ^2^	−0.08	−0.09	−0.63	0.13	0.35	−0.52	0.43	0.49	0.08
RMSE	0.15	0.13	0.17	0.14	0.1	0.17	0.11	0.09	0.13
nRMSE	43.91%	47.38%	41.02%	39.41%	36.55%	39.62%	31.92%	32.47%	30.80%
PH									
	VCM-1 vs. RCM			VCM-4 vs. RCM			VCM-8 vs. RCM		
	All-stage	48 DAS	70 DAS	All-stage	48 DAS	70 DAS	All-stage	48 DAS	70 DAS
*R* ^2^	0.14	−0.2	−0.31	0.73	0.63	0.57	0.85	0.81	0.72
RMSE	31.74	38.74	22.67	17.81	21.62	12.92	13.15	15.37	10.48
nRMSE	17.85%	24.34%	11.54%	10.02%	13.59%	6.58%	7.40%	9.66%	5.33%
PAI									
	VCM-1 vs. RCM			VCM-4 vs. RCM			VCM-8 vs. RCM		
	All-stage	48 DAS	70 DAS	All-stage	48 DAS	70 DAS	All-stage	48 DAS	70 DAS
*R* ^2^	−0.62	−0.03	−3.65	−0.36	0.37	−2.03	0.17	0.65	−1.33
RMSE	2.31	1.82	2.94	2.12	1.42	2.38	1.66	1.06	2.09
nRMSE	50.23%	48.86%	53.74%	40.02%	38.08%	43.41%	35.00%	17.24%	37.43%
COV									
	VCM-1 vs. RCM			VCM-4 vs. RCM			VCM-8 vs. RCM		
	All-stage	48 DAS	70 DAS	All-stage	48 DAS	70 DAS	All-stage	48 DAS	70 DAS
*R* ^2^	−0.39	0.29	−3.83	−0.14	0.53	−2.06	0.25	0.71	−1.52
RMSE	0.41	0.33	0.51	0.37	0.27	0.4	0.3	0.21	0.37
nRMSE	44.51%	41.92%	48.96%	36.44%	34.28%	38.98%	32.69%	26.61%	35.35%

## Discussion and Conclusion

### Differences in light interception between RCM and VCMs

Differences in light interception in plant canopies are often caused by structural differences [13]. Variations in plant canopy structure, such as leaf aspect ratio, leaf inclination angle, and canopy coverage, can lead to large differences in light interception [53,54]. Several studies have shown that plant structural parameters can be used as variables to uniquely explain variations in light interception across the canopy scale [49,55]. However, VCMs have often been used instead of RCM to study canopy light interception in the field [29,30], and their accuracy remains unknown. The study confirmed substantial differences in light interception between the RCM and the 3 types of VCMs. The difference in light interception decreased as the number of replicated plants used in the virtual canopy increased, but the difference remained substantial even when using a VCM consisting of 8 plant replicates. These results suggest that the use of an RCM can greatly improve the accuracy of 3D canopy light interception calculations in the field.

The structure of the crop canopy is a crucial factor affecting its light interception capability. The thickness and density of the canopy directly influence the transmission of light. A thicker, denser canopy can intercept more sunlight, thereby enhancing the efficiency of photosynthesis [56]. However, an excessively dense canopy may prevent light from penetrating lower leaves, thereby limiting the overall photosynthetic capacity. The distribution of leaf area and leaf angle is the most important structural parameter affecting light interception. The distribution of leaf area density affects the spatial distribution of the shading leaf area. For corn, plant height and ear height increase with the increase in plant density and LAI, while the leaf area per plant shows a logarithmic decrease [57]. The part of light intercepted by the plant increases with an increase in plant density, but the light attenuation coefficient linearly decreases [57]. Although plants with larger leaves have more mutual shading effects, under the same light conditions, their larger leaf area captures more light than smaller leaf crowns [58]. Smaller leaf canopies demonstrate a higher light penetration efficiency and a higher unit leaf area light interception rate. The direction of the leaves affects not only the spatial distribution of the shading leaf area but also the distribution of light intensity on the leaf surface. If the leaves are vertical, they can intercept more light in the morning and evening, while they may not capture enough light when the sun is directly overhead [59]. Therefore, plants adjust the angle of their leaves to maximize light interception.

The notable difference in DLI between VCM-1 and RCM (*R*^2^ = 0.45, rRMSE = 20.22%, Table 1) is due to the fact that VCM-1 is replicated from a single plant, resulting in a different canopy structure (Table 2) compared to RCM. VCM-1 cannot reflect the interactions between plants in the real environment [60]. VCM-8 has a canopy structure closer to RCM (Table 2), resulting in a lower light interception difference (*R*^2^ = 0.68, Table 1). However, the rRMSE is still greater than 10% (rRMSE = 15.48%, Table 1) as the VCM-8 canopy density still differed compared to the RCM (Fig. 7A and D). There were substantial differences in the 2D traits PAI of VCM and RCM, proving that one of the reasons for the light interception differences between VCM and RCM is the difference in their leaf areas. The 3D traits COV is a comprehensive trait that takes into account the combined effects of leaf area and leaf angle [49]. Although there are substantial differences in their COV, it is unclear how much of the light interception differences between VCM and RCM are due to changes in leaf angle. Based on the FSPM model, by fixing PAI and only changing the leaf angle to calculate the light interception differences between the VCM and RCM, it is more effective in distinguishing the impact of leaf angle changes on the light interception of VCM and RCM. The estimated PH of the VCM is lower than the RCM (Table 1) because the replicated plant chosen for the VCM is often not the tallest among the stand. It was also found that the difference in light interception between RCM and VCMs was greatly smaller in the early stage (48 DAS) than in the late stage (70 DAS). This occurs because the changes in the canopy structure of the VCMs and the RCM are more pronounced in the later stages than in the early stages, resulting in marked variations in light interception.

### Realistic 3D canopy acquisition in the field

Capturing a realistic 3D canopy structure of plants is crucial for understanding their morphology and structure, which, in turn, helps to study their growth, development, and response to environmental factors [26]. It also facilitates the identification and quantification of key plant structures such as leaves, stems, roots, and flowers, which are important for plant breeding [61]. LiDAR and SfM-MVS were 2 of the most commonly used techniques for obtaining 3D canopy structure information [31,62]. Although LiDAR provides high accuracy, it is an expensive technology that lacks flexibility in field data collection. SfM-MVS, on the other hand, is a low-cost alternative that only requires an RGB camera. The combination of a drone with SfM-MVS offers a more flexible and affordable solution and shows great potential for 3D canopy data collection in the field. In this paper, the UAV CCO route was used to obtain 3D point clouds of maize canopies over a large field area, accurately extracting organ-scale features such as leaf length and width for different layers. Incomplete leaf reconstructions (Fig. 5C and D) were observed due to leaf shading. The CCO route, which captures images from a 360° perspective, enhances the acquisition of data from the obscured portions of the canopy. This technique effectively reconstructs the maize canopy during its early to mid-stages due to minimal canopy occlusion. However, full reconstruction of the maize canopy in its later stages remains a challenge. Through testing, it has been found that capturing 40 images in each single route of CCO to achieve a 90% intra-circle overlap can successfully reconstruct the late-stage corn canopy. In this study, segmentation and estimation of organ-scale traits were performed manually. There have been several proposed algorithms for segmenting organ-scale traits from 3D point clouds of single plants [63,64]. However, there remains a relative scarcity of organ-scale segmentation algorithms for field crops. Current high-throughput plant breeding efforts using drones focus on plot-scale traits, including plant height [65] and canopy coverage [66] There is an urgent need for drones to be used for organ-scale breeding in the field, producing accurate 3D canopy models and high-throughput estimates of organ-scale traits, which would accelerate the plant breeding process [67,68].

This study did not perform a sensitivity analysis to provide a specific value that would allow a small subset of the canopy to be representative of the entire canopy. This is because our results demonstrate that the more plants that replicate the canopy, the more they are representative of the entire canopy. Our proposed CCO method can obtain the complete canopy 3D structure efficiently and precisely. Therefore, we conclude that reconstructing the complete 3D canopy based on CCO and calculating light interception is more efficient and accurate than the traditional method of replicating plots to calculate light interception. Despite our concerted efforts to provide a comprehensive study, we acknowledge that our current research has its limitations. Although the study has substantiated the differences in light interception between the VCM and the RCM, we have not delved deeper into analyzing the factors causing these differences, such as the impact of leaf angles. This limitation arises because, despite our study reconstructing the precise 3D canopy structure of corn in the field based on UAVs, the extraction of leaf angle information remains a considerable challenge. To obtain this leaf angle information, we first need to segment the corn point cloud of a population into point clouds of single corn plants, which are then further divided into point clouds of individual leaves. In the current research, accurately separating complete individual corn plants from a 3D point cloud of a population is still challenging due to the overlapping of leaves. Existing studies capable of segmenting individual plants from a corn population have been conducted under the premise of sparse artificial planting. We are currently conducting research in this area and may be able to achieve this goal in the near future.

The accuracy of the 3D structure of the plant affects the accuracy of light interception. An RCM reconstructed by the UAV CCO method was compared with 3 types of VCMs for light interception. The VCMs were replicated from different numbers of realistic plants. The DLI of the 3 VCMs differed noticeably from the RCM, and this difference decreased with the number increase of plants used to replicate the virtual canopy. The results confirm that the light interception calculated by the VCMs is not accurate and that RCM needs to be constructed in the field to calculate light interception more accurately, especially in the later growth stages of plant.

## Data Availability

The data used in this study are freely available. Anyone who wants to use the data can contact the corresponding author Y.M. The author is affiliated with the College of Land Science and Technology, China Agricultural University, Beijing 100193, China (e-mail: yuntao.ma@cau.edu.cn).
